# The dynamics of DNA methylation, histone methylation and acetylation during oocyte aging in mammalian species and possible interventions to regulate them

**DOI:** 10.1007/s10815-025-03577-4

**Published:** 2025-07-14

**Authors:** Saffet Ozturk

**Affiliations:** Department of Histology and Embryology, Akdeniz University School of Medicine, Campus, 07070 Antalya, Türkiye

**Keywords:** DNA methylation, Histone methylation, Histone acetylation, Oocyte aging, Epigenetic regulation

## Abstract

Oocyt e development from non-growing to metaphase II (MII) stages is largely dependent on timely and correctly controlling gene expression. During the process of biological or postovulatory aging, the epigenetic mechanisms, particularly DNA methylation, histone methylation, and acetylation, exhibit notable changes in oocytes at various stages of development. These changes mainly result from altered expression of the related catalytic enzymes. In this review, changes in DNA methylation, histone methylation, and acetylation marks and expression of the acting enzymes in aging mammalian oocytes have been comprehensively evaluated in the light of existing studies. Potential interactions between these epigenetic mechanisms are also discussed. Finally, possible interventions to regulate them in order to mitigate the loss of female fertility in the later periods of the reproductive lifespan are reviewed.

## Introduction

In recent decades, the majority of couples have chosen to postpone having children due to socioeconomic reasons and career planning [1]. The average age of women at first birth has increased by 2–4 years over the last 20–30 years. In other words, the age at which women give birth for the first time is over 30 in many countries. The birth rate of human ART patients drops from 19.2% at age 38 to 5.1% at age 43 [2]. This decrease may be due to the negative correlation between fertility and chronological age in mammalian species [3]. Consequently, the window of opportunity for reproduction is reduced as a result of postponing childbearing, necessitating the involvement of medical professionals.

During maternal aging, biological functions of the ovaries gradually decline, defined as reproductive aging. When biological aging is greater than chronological age, this is known as accelerated aging and is associated with poor reproductive outcomes. In the opposite version, there is an age deceleration, which shows better health conditions. Defects in meiotic resumption, reduced oocyte quality and quantity, and decreased antral follicle count are the main hallmarks of maternal aging. As the ovaries age, the number of follicles produced in each cycle decreases, leading to a reduction in ovarian size [4]. In addition, while the levels of the reproductive hormones estrogen and progesterone decrease [5, 6], the production of follicle stimulating hormone (FSH) and luteinizing hormone (LH) by the pituitary gland increases with advancing maternal age [7].

In an elegant study, oocyte transfers from young donors to older women undergoing in vitro fertilization (IVF) have improved reproductive performance compared to oocyte transfers from age-matched older women to recipients [8]. According to the guidelines of the American Society for Reproductive Medicine (ASRM) 2024, oocyte donors should preferably be between the ages of 21 and 34, as oocyte translation efficiency decreases with age [9]. As women age, the functional quality of their oocytes declines towards the onset of menopause. Possible causes of poor oocyte quality include reduced ooplasm quality, mitochondrial dysfunction, altered chromatin structure, and defects in the meiotic machinery [10, 11].

Genomic instability, telomere attrition, stem cell depletion, chronic inflammation, and epigenetic changes are the major hallmarks of oocyte aging [12, 13]. In short, the epigenome is defined as the many chemical modifications on chromatin that regulate transcriptional activity in a time-dependent manner. Compared to genetic changes in the DNA itself, epigenetic changes that impact DNA packaging, stability, and expression are reversible, self-perpetuating, and heritable, and intracellular signaling pathways and potential changes in cellular and environmental conditions can affect the epigenome. Covalent and non-covalent modifications in DNA and associated histone proteins contribute to the regulation of gene expression, chromatin structure, DNA packaging, and genome stability [14, 15]. In the following parts of this review, after introducing DNA methylation, histone methylation, and acetylation mechanisms, their potential relationship with oocyte aging will be discussed in detail.

## DNA methylation and demethylation dynamics in aging oocytes

DNA methylation is briefly defined as the addition of a methyl group to cytosine residues located in cytosine phosphate-guanine (CpG) dinucleotides and non-CpG sites. In this way, gene expression is controlled in a time-dependent manner in cooperation with other epigenetic mechanisms. Specifically, high levels of DNA methylation at CpG regions of gene promoters, referred to as hypermethylation, result in gene repression and chromatin compaction, while its low levels, referred to as hypomethylation, are associated with gene activation. In fact, there are two types of DNA methylation processes, known as maintenance and de novo. In the maintenance methylation, DNA methyltransferase 1 (DNMT1) plays a key role in adding methyl groups to hemi-methylated DNA strands during DNA replication. In the process of de novo methylation, the DNA methyltransferases DNMT3A and DNMT3B work with DNMT3L to add methyl groups to unmethylated DNA strands.

As is known, human oocytes undergo physiological aging at the age of ≥ 35 years because of suboptimal reproductive environments that result in decreasing oocyte quality and developmental potential of early embryos [16]. Research on human oocytes are restricted in the majority of countries due to ethical and legal issues and limited availability [17]. Thus, most studies have been performed on animal models, especially porcine, bovines, and murine, because their oocytes show similarities in the aspects of transcription-related hyper- and hypomethylated domains, but there are differences in the context of transcription-related other methylated regions and active DNA demethylation by TETs with humans [18]. In other words, as these mammalian species exhibit parallels with humans regarding follicular selection, embryo cleavage, blastocyst formation, and gestation, they are being used as suitable animal models [19].

### Growing oocytes

In aging mouse ovaries, our recent study showed that both mRNA and protein levels of the *Dnmt1*, *Dnmt3a*, and *Dnmt3l* genes, as well as global DNA methylation [shown by 5-methylcytosine (5-mC) staining], decreased in older ages (52-, 60-, and 72-week-old) compared to the young ages (5- and 6-week-old) [20]. However, *Dnmt3b* expression increased in the ovaries from older ages. These changes may result from their altered expression in oocytes and granulosa cells of the follicles from primordial to antral stages at the later ages. In the rat ovaries at the ages of 6, 12, and 24 months old, whereas DNMT1 and DNMT2 proteins and their mRNA levels decreased with aging ovaries, both DNMT3A and DNMT3B expression increased along with ovarian aging [21]. Similar to most DNMTs expression patterns, the *Tet1*, *Tet2*, and *Tet3* gene expression at both mRNA and protein levels were reduced significantly in aging mouse ovaries [22]. Overexpression of TET1 protein was found to induce proliferation and even suppress apoptosis in aging human ovarian cell lines [22]. Given the reduction in global DNA methylation observed in aged ovaries, it is plausible that TETs expression may be diminished in order to prevent further DNA demethylation. The rising expression of de novo methyltransferases may facilitate the restoration of methylation patterns in aged cells, possibly to ensure the continuation of cellular lifespan.

**Fig. 1:**
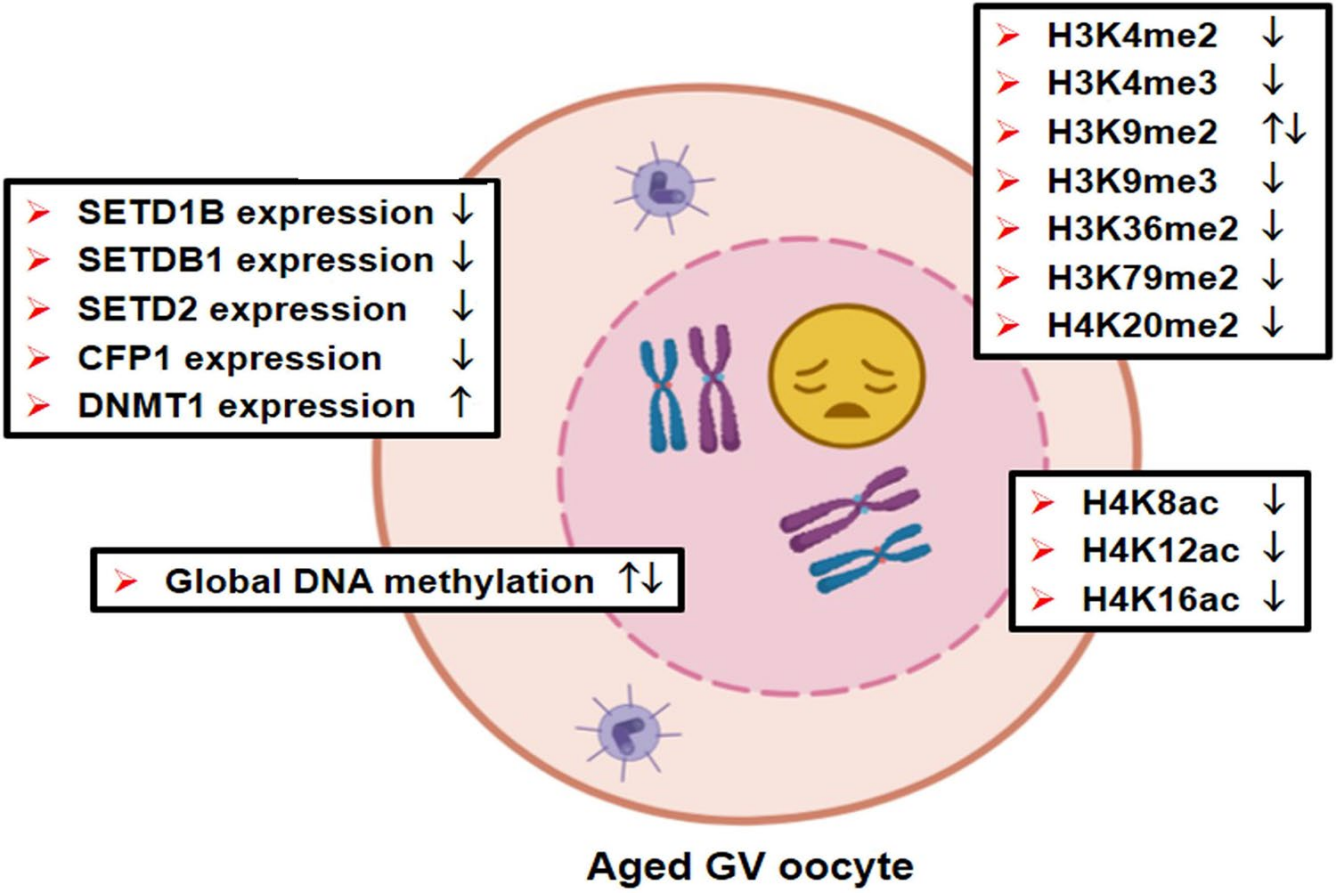
The relative levels of DNA methylation, histone methylation, and acetylation and some related enzymes in aged germinal vesicle (GV) oocytes. While histone methylation-related protein levels decrease, maintenance DNA methylation-related DNMT1 levels increase in aged GV oocytes. Furthermore, most histone methylation and acetylation on H3 and H4 decrease in aged GV oocytes. The icons represent either a decrease in expression (↓) or an increase in expression (↑) in the aged group when compared to controls. Notably, the coexistence of ↓ and ↑ is indicative of disparate findings. This figure was created utilizing the BioRender program (BioRender; Toronto, Canada). me, methylation; ac, acetylation

Global DNA methylation, as measured by 5-mC staining, was found to be increased in fully grown GV oocytes from aged mice at 69–70 weeks compared to young mice at 10–13 weeks [16, 23] (Fig. 1). Similarly, DNA methylation in the upstream control element and promoter of ribosomal DNA (rDNA) was increased in GV oocytes from older women (33–39 years) when compared to the GV oocytes from young women (26–32 years) [24]. Consistent with increasing global DNA methylation, DNMT1 protein levels enhanced in the GV oocytes from aged mice [16]. On the other hand, Castillo-Fernandez et al. (2020) reported that global DNA methylation decreased in GV oocytes obtained from mice at 44–54 weeks compared to young mice at 12 weeks [25].

In the later ages, single-cell RNA-sequencing and single-cell whole-genome DNA methylation sequencing on the GV oocytes obtained from young (8-week-old) and aged (18-month-old) revealed that there were also significant changes in the DNA methylation of some metabolism-related genes including mitochondria-associated genes [26]. The CpGs in the *DENND1A* (having a role in clathrin-mediated endocytosis) intron exhibited hypermethylation in the immature oocytes obtained from reproductively aging cattle [19]. However, both downstream and upstream of the sirtuin 1 (*SIRT1*) gene showed hypomethylation in these oocytes [19]. All these results showed that while global DNA methylation decreases in early old ages, it increases in late old ages. This situation may result from the endeavor of aging oocytes to protect themselves from unfavorable effects of aging through controlling DNA methylation levels and possibly its genomic distribution.

In in vitro culturing (IVM) procedure, 48 h IVM (extended culturing) of bovine oocytes and the resulting embryos showed differently methylated CpG sites in the *DNMT3L* gene when compared to the oocytes experienced 24 h IVM (control) protocol [27]. Extended IVM caused decreased transcript level of the imprinted *IGF2R* gene as well as reduced transcript levels of *PRDX1*, *DNMT3A*, and *BCLXL* in 4- to 8-cell embryos obtained from in vitro-aged oocytes [27]. Altered expression of these genes may result from changes in their DNA methylation profiles and distribution, because DNMT3L works as a catalytic activator of DNMT3A and DNMT3B.

In the aging mouse ovaries, TET1, 2, and 3 expression at mRNA and protein levels decreased [22]. Expectedly, while the 5-mC staining increased, the 5-hydroxymethylcytosine (5-hmC) staining decreased in the mouse ovaries from the aged group at 6–8 months old when compared to the young one at 6–8 weeks old [22]. Having an intrinsic auto-regulatory mechanism of TET dioxygenases contributes to regulating its enzymatic activity so that DNA methylome reprogramming is strictly controlled during oocyte development [28]. Thus, the mouse oocyte epigenome is immediately protected from untimely undergoing oxidative demethylation [28]. It is possible that the control mechanism on TETs may disrupt during ovarian aging, which could result in altered DNA methylation dynamics.

### Mature oocytes

**Fig. 2:**
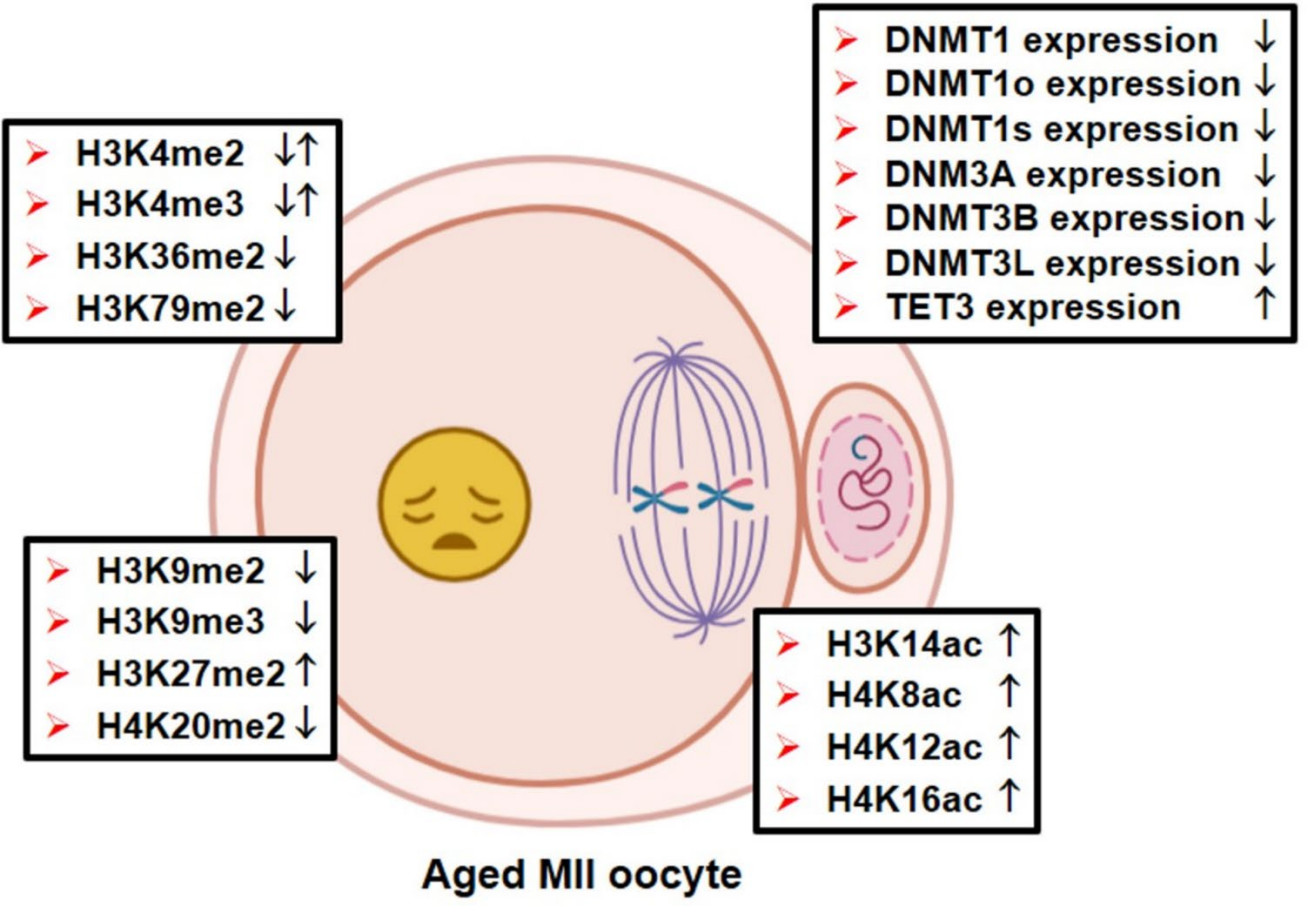
The relative relevels of DNA methylation, histone methylation, and acetylation and some related enzymes in aged metaphase II (MII) oocytes. While DNA methylation–related proteins (including different types of DNMTs) levels decreased, except for TET3 expression, transcriptional activation (H3K4me2, H3K4me3, H3K36me2, and H3K79me2), or repression (H3K9me2, H3K9me3, H3K27me2, H3K36me2, and H4K20me2) related histone methylation marks decreased in aged MII oocytes. However, there was an increase in the histone acetylation marks, including H3K14ac, H4K8ac, and H4K12ac. The icons represent either a decrease in expression (↓) or an increase in expression (↑) in the aged group when compared to controls. Notably, the coexistence of ↓ and ↑ is indicative of disparate findings. This figure was created utilizing the BioRender program (BioRender; Toronto, Canada). me, methylation; ac, acetylation

As in GV oocytes, in MII oocytes, global DNA methylation detected by 5-mC staining decreased in older mice (35–40 weeks) compared to younger mice (6–8 weeks) [29]. The observed decrease appears to be due to a reduction in the expression of DNMT1, DNMT3A, DNMT3B, and DNMT3L in the MII oocytes of older mice [29]. Another possible explanation is that changes in the DNA methylation patterns within their gene control regions may lead to a reduction in the protein levels. In line with this prediction, expression of the *Dnmt1o*, *Dnmt1s*, *Dnmt3a*, *Dnmt3b*, and *Dnmt3l* mRNAs decreased in the MII oocytes from older females at 42–45 weeks old (versus young ones at 5–6 weeks old) [30] or 36 weeks (versus young ones at 4–5 weeks old) [31]. As expected, TET3 protein level, a main demethylase in the germline, increased in the MII oocytes from mice undergone natural (18-week old) or accelerated aging [32]. These results on mice indicate that, in order to reduce global DNA methylation levels in MII oocytes during aging, there is not only a decline in DNMT levels but also TETs appear to be elevated (Fig. 2). On the other hand, Paczkowski et al. (2015) found no change in the expression of imprinting-related genes, including *Snrpn*, *H19*, *Mest*, *Peg3*, or *Igf2r*; however, there was an increase in *Kcnq1* gene expression in MII oocytes from mice at 15 months of age compared to those at 5 weeks of age [33]. How imprinting sites are protected and the potential reasons for changed expression of the *Kcnq1* gene during aging is the goal of future studies.

In aged porcine MII oocytes, DNA hypermethylation was observed in the neuronatin (*NNAT*, being involved in the regulation of ion channels) gene, which resulted in declining its expression [34]. As a result of decreased expression of NNAT, glucose transporter mechanisms were disturbed, and thus, oocyte quality was reduced [34]. In aged (8–11 years old) bovine oocytes, only 9 of 1044 alleles (< 1%) showed abnormal methylation (> 50% of CpGs having abnormal methylation status) in the *H19* and *DNMT3L* genes when compared to the younger groups, prepubertal (9–12 months old) and mature (3–7 years old) [35]. The results from the mouse, porcine, and bovine studies revealed that there are differences in DNA methylation dynamics in aging oocytes, possibly due to having species-dependent distinct epigenetic control mechanisms during oocyte maturation processes.

In the postovulatory aging of mouse oocytes, while the *Snrpn* gene enables protection of the complete methylation pattern at 13 and 21 h post hCG injection, there was methylation lost in some oocytes at 29 h post hCG injection [36]. For the *Peg1* gene, while Liang et al. (2008) found hypermethylation [36], Imamura et al. (2005) revealed loss of methylation in the oocytes throughout postovulatory aging [37]. The other studies reported that although postovulatory oocyte aging led to a decrease in reproductive outcomes and longevity of offspring, no disruption was noted in the methylation imprints of oocytes from viable offspring [38, 39]. These differences may result from using distinct time points for oocyte aging or evaluating different regions of the target genes.

The in vitro culturing was found to cause hypermethylation in the *Peg1* (also known as *Mest*) gene, whereas prolonged in vitro culturing of mouse MII oocytes resulted in demethylation of this imprinted gene [37]. Additionally, the fully methylated alleles at CpG and non-CpG sites in the oocytes underwent demethylation in the 2-cell embryos and blastocysts [37]. Additionally, in the in vitro matured GV and MII oocytes, both DNMT3A and DNMT3B proteins were localized around the chromosomes at GV breakdown and pro-MI stages from young mice (7–8 weeks old); however, these proteins resided in the cytoplasm in aged mice (40–47 weeks old) [40]. As there is no change in the cytoplasmic levels of these DNMTs, the efficiency of cytoplasm-to-nucleus trafficking may be associated with adjusting DNA methylation profiles. The precise mechanisms by which DNMTs are transferred to the nucleus should be evaluated in aging oocytes to understand the molecular background of DNA methylation establishment in certain genes.

In an investigation of accelerated oocyte aging [induced by treatment with 4-vinylcyclohexene diepoxide (VCD)], Qian et al. (2015) conducted a comparative analysis of modified cytosines [5-mC, 5-hmC, and 5-formylcytosine (5-fC)] and *Tet* expression between the aged and young oocytes obtained from 28-day-old mice [32]. While the 5-mC level decreased in the aged group, the 5-hmC and 5-fC levels increased in the aged group compared with the young group [32]. As expected, the acceleration of aging resulted in an increase in the *Tet3* gene expression when compared to the young group. However, no change was noted for the *Tet1* and *Tet2* mRNA levels [32]. Age acceleration also showed a negative correlation with the antral follicle count, AMH levels, total numbers of mature and fertilized oocytes, and two-pronuclear oocytes [41]. In other words, it was associated with decreased ovarian reserve and oocyte quality in women. All these changes occurring during accelerated aging in oocytes may be associated with disrupted DNA methylation patterns and thereby altered gene expression.

As a result, the accelerated, in vitro, and postovulatory aging has been observed to induce alterations in DNA methylation patterns in both GV and MII oocytes, in addition to influencing the localization and expression levels of DNMTs and TETs. These changes are likely the result of a reduction in the expression and altered localization of DNMTs as well as decreased levels of TETs in these oocyte stages. A deeper understanding of the mechanisms regulating expression of these enzymes and their subcellular localizations may facilitate the development of strategies to preserve DNA methylation maintenance in oocytes during maturation at later reproductive ages.

## Histone methylation during oocyte aging

Histone modifications are known as chemical changes to amino acid residues of histones, generally localized to the N-terminal tails extending from globular nucleosomes. Histone methylation contributes to transcriptional activation or repression based on its levels, amino acid residues, and locations in chromatin. While methylation of H3K4, H3K36, and H3K79 induces transcriptional activity, the H3K9, H3K27, and H4K20 methylations inhibit transcription [42, 43]. It was reported that, from a general perspective, histone lysine trimethylation increased with age in 12- and 17-month-old mouse ovaries compared to 3-month-old ovaries [44]. However, dimethylation of lysine residues in the ovaries of 12-month-old mice decreased when compared to 3-month-old mice. These findings highlight that trimethylation and dimethylation in lysine residues show dynamic differences in aged ovaries, which may result from altered expression of their related enzymes. An evaluation of these changes in the context of transcriptional reflection is also warranted.

The levels of the histone residues including H3K9me3, H3K36me2, H3K79me2, and H4K20me2 decreased in the GV and MII oocytes of 11-month-old mice when compared to the young 2-month-old ones [45] (Figs. 1 and 2). While GV oocytes from aged mice with surrounded nucleolus chromatin conformation had lower H3K4me2 levels, the H3K9me2 level decreased in GV oocytes with non-surrounded nucleolus conformation from aged females. These changes are convenient with the transcriptional activity of these GV oocyte substages. The H3K4me2 and H3K9me2 levels were also reduced in the MII oocytes from the aged group [45]. The same group also reported in their later study that an increase in H3K9me2 was observed in the fully grown GV oocytes of aged mice (69–70 weeks) compared to young mice (10–13 weeks) [16]. In contrast, the in vitro preovulatory aging process resulted in a reduction of H3K9me3 levels in murine oocytes, accompanied by additional alterations such as chromosome misalignment and aberrant spindle conformation [46]. These studies suggest that MII oocytes express these histone methylation marks independent of transcriptional control and the types of aging.

An interesting study by Shao et al. (2015) demonstrated that there was a decrease in H3K4me2/3 levels in the percentage of GV oocytes from aged mice (42–44 weeks old) compared to young mice (6–8 weeks old), but more MII oocytes from the aged group had higher H3K4me2/me3 staining intensities [47]. H3K4me3 is known to be associated with meiotic resumption, spindle assembly, and chromosome alignment as well as transcriptional activation in the promoter regions of genes [48], whereas H3K9me2 is associated with facultative heterochromatin (silent DNA) formation [49, 50]. While no difference was observed among GV oocytes with NSN type, a decrease in H3K4me3 level was noted in GV oocytes with SN type and MII oocytes of aged mice in comparison to those of young mice [51]. The in vitro-aged (24 h) porcine MII oocytes exhibited a reduction in H3K4me3 levels when compared to the young MII oocytes [51] (Fig. 2). In the context of transcriptional control, the role of H3K4me2/3 during oocyte maturation appears to be subject to disruption, as evidenced by unexpected fluctuations in H3K4 methylation levels in GV and MII oocytes with advancing age.

All the aforementioned findings indicate a decline in the transcriptional activation- or repression-related H3 and H4 methylation marks in aged GV and MII oocytes. Alterations in histone methylation in oocytes with a SN-type nucleolus may affect typically appearing cessation of transcription, while changes in oocytes with an NSN type could influence the deposited levels of maternal transcripts, which are translated during oocyte maturation and early preimplantation embryo development. It is crucial to acknowledge that histone methylation alterations observed in aged MII oocytes may potentially originate from the preceding GV oocytes.

In addition to the effects of biological aging, prolonged in vitro culturing of porcine MII oocytes (24 h after 44 h in vitro maturation) resulted in elevated levels of H3K4me2 and H3K27me2 [52]. It is noteworthy that administration of melatonin contributed to safeguarding histone modifications in oocytes subjected to prolonged culturing [52]. In a previous study, Trapphoff et al. (2016) found a decreased pericentromeric H3K9me3 level in MII oocytes which underwent aging for 24 h post-ovulation [53]. The H3K4me2/me3 levels were observed to increase in MII oocytes during postovulatory aging at 6 and 12 h [54]. Further studies on oocytes experiencing postovulatory aging should be evaluated to determine exact effects of in vitro and in vivo conditions. A limited number of studies suggest that postovulatory aging may affect transcription-related histone methylation marks especially in transcriptionally silent MII oocytes.

The expression of EZH2, which is responsible for the production of H3K27me3, was reduced in MII oocytes derived from aged mice (42–45 weeks old) when compared to those derived from young female mice (5–6 weeks old) [55, 56]. The expression of KMTs including SETD1B (H3K4me1/2/3), SETDB1 (H3K9me3), SETD2 (H3K36me3), and CFP1 (H3K4me3) protein levels playing roles in creating transcription-related histone methylation marks in parenthesis decreased in the follicles, GV oocytes, and granulosa cells as well as in the germinal epithelial and stromal cells in the aged groups when compared to the young groups [57] (Fig. 1). However, the protein levels of KMT5B and KMT5C, both of which are involved in H4K20 methylation, elevated in the ovaries of 17-month-old mice when compared to those of 3-month-old mice [44].

As a result, while a limited number of studies have evaluated KMTs expression in aging oocytes, the results indicate that there are changes in their levels, which are accompanied by altered histone methylation marks. In addition to KMTs, it would be beneficial to examine demethyltransferases in aging oocytes to determine the underlying molecular mechanisms responsible for altered histone methylation levels.

## Histone acetylation during oocyte aging

Histone acetylation is primarily involved in transcriptional control and chromatin remodeling. The levels of acetylation marks and related enzymes show dynamic changes in oocytes during biological and postovulatory aging. The H4K8ac, H4K12ac, and H4K16ac levels decreased in the old GV oocytes, especially with surrounded nucleolus from the aged mouse group (10–11 months old) when compared to the young group (3 months old) [58] (Fig. 1). The GV oocytes from aged females at 10 and 15 months showed 83% and 53% decreases in H4K12ac expression, respectively, compared to those from young 2-month-old [58]. Since acetylation is known to control transcriptional activation, its reduced levels in GV oocytes from older mice may result in premature transactional repression.

In MII oocytes, H4K12 acetylation that occurs during meiotic resumption was maintained in old mice (11, 13, and 15 months), whereas it was completely deacetylated in young oocytes [58, 59]. Increased H4K8ac was found in MII oocytes from old (10 months old) compared to young (3 weeks old) mice, indicating incomplete deacetylation [59]. However, Manosalva and Gonzalez (2009) determined no change in H4K8ac in MII oocytes of the aged group (10–11 months old) compared with the young (2 months old) group (Fig. 2). The observed discrepancy may be attributed to the use of disparate mouse strains and the presence of methodological discrepancies. The maintenance/alteration of these histone acetylation marks in aging MII oocytes indicates that the dynamic control of these marks by specific acetyltransferases and deacetylases is impaired.

High levels of H4K12ac in MII oocytes from aged murine females (10 months or 35–40 weeks old) were associated with abnormal chromosome distribution [59, 60]. H4K12ac was also retained in MII oocytes from women > 31 years old compared to the oocytes from young women (21–30 years old) [61]. Herein, a positive correlation was demonstrated between the accumulation of this modification and increased chromosomal aberrations in the oocytes of older women [61]. In other words, a defect in deacetylation of H4K12 was found to be associated with chromosome misalignment, suggesting that H4K12ac accumulation may lead to disruption in control of the genes having roles in chromosome segregations. In a recent study, He et al. (2023) reported that H4K12ac level increased in MII oocytes of aged mice when compared to young mice [51]. An intervention to reduce H4K12ac level in MII oocytes undergoing biological aging may contribute to decreasing chromosomal abnormalities.

In the mouse model of postovulatory aging, although there were no significant changes in H3K9ac, H3K14ac, H4K5ac, H4K12ac, and H4K16ac in oocytes obtained 1 h after hCG treatment, fluorescent staining of H4K8ac and H4K12ac increased in oocytes after 19 h of hCG injection [62]. After 24 h of hCG injection, there was an increase in the H3K14ac levels in the oocytes [62]. Chromosome misalignment and dispersal increased in MII oocytes undergone postovulatory aging (34 h post-hCG) when compared to control oocytes (14 h post-hCG) [63]. These effects may be associated with enhanced levels of H3K14ac and H4K16ac in aged MII oocytes as well as decreased H3K9 methylation [63]. Similarly, Liu et al. (2009) observed an increased expression of H3K14ac and H4K12ac levels in the mouse oocytes during postovulatory aging [64].

In porcines, an enhancement in the acetylation of H4K12 was found in the mature oocytes during in vitro postovulatory aging [65]. In addition to spindle abnormalities and misaligned chromosomes, an increased level of H4K12ac was observed in MII oocytes that experienced 24 h of postovulatory aging [53]. Similarly, a recent study demonstrated that in vitro-aged (24 h) porcine MII oocytes exhibited a higher level of H4K12ac compared to control oocytes [51]. H3K9ac levels were further found to be increased in postovulatory aged oocytes [54]. The KIF15 (also known as HKLP2) belongs to the kinesin-12 superfamily and is involved in Golgi apparatus and vesicle-related transport as a cytoplasmic motor protein. In a recent study by Yin et al. (2024) found KIF15 expression in porcine MII oocytes during maturation, and it was localized dependent on microtubule dynamics [66]. KIF15 also plays a role in regulating HDAC6-related microtubule stability for spindle organization during meiosis. Decreased KIF15 expression in postovulatory aged porcine oocytes may lead to their reduced maturation competence [66].

Taken together, all these studies have demonstrated that histones of the maternal genomes of mammalian species undergo deacetylation during meiotic resumption and progression from GV to MII stages. Also, some histone types show an increased acetylation in both in vitro and in vivo aging conditions. An expected rise in histone acetylation levels has been observed in mammalian oocytes with advancing age (Fig. 2), which may disrupt the strict regulation of gene expression during oocyte maturation and subsequent early embryo development.

The expression of histone deacetylase 1 (HDAC1) was found to decrease significantly in aged mouse and porcine oocytes in both maternal and postovulatory aging, which resulted in a change in the level of H4K12ac [51]. Melatonin treatment prevented the loss of HDAC1 protein and partially corrected H4K12ac status in aged oocytes [51]. Accordingly, expression of HDAC2, which plays a role in the deacetylation of lysine residues at the N-terminal regions of H2A, H2B, H3, and H4 histones, decreased in MII oocytes from aged mice (42–45 weeks old) when compared to oocytes from young female mice (5–6 weeks old) [55, 56]. The mRNA and protein levels of SIRT1, known as an NAD + -dependent deacetylase, were also downregulated in 6- and 12-h postovulatory aged oocytes compared to fresh oocytes [54]. Importantly, overexpression of SIRT1 in postovulatory aged oocytes resulted in decreased H3K9ac accumulation, in addition to reduced age-related morphological changes and ROS abundance, as well as maintained normal spindle morphology and attenuated age-related mitochondrial abnormalities [54]. These findings suggest that reestablishing related enzyme activities may contribute to maintaining equilibrium between histone acetylation levels in aged oocytes because maternal and postovulatory aging lead to decreasing expression of certain histone deacetylases by as yet undefined mechanisms.

Histone deacetylase 3 (HDAC3), which has been demonstrated to facilitate assembly of the meiotic apparatus in mouse oocytes, exhibited a notable decline in GV oocytes derived from aged mice [67]. As expected, its overexpression in old GV oocytes contributed to preventing spindle and chromosome disorganization as well as declining aneuploidy rates [67]. Given that HDAC3 is involved in deacetylation of histones H3 and H4, a reduction in HDAC3 levels in aging oocytes may result in the disruption of oocyte nuclear maturation-related gene expression. Maintaining optimal levels of HDAC3 appears to be imperative for the successful extraction of competent oocytes from aged mammalian species, particularly in murine models. Further studies on humans may provide insights into the significance of HDAC3 in the acquisition of high-quality mature oocytes in ART centers.

Overall, significant changes in histone acetylation and deacetylation levels have been observed in oocytes undergoing maternal or postovulatory aging, at either GV or MII stage. These changes may be attributed to altered expression or activity of the associated enzymes. It would be beneficial for future studies to investigate the underlying mechanisms of these changes in histone acetyltransferases and deacetylases during aging. The reproductive hormones, especially estrogen and progesterone, and other microenvironmental changes may be potential factors that can be associated with these changes.

## Intervention to epigenetic mechanisms of aging oocytes

Given that epigenetic modifications are reversible, they can be targeted through the intervention of epigenetic regulators for the purpose of attenuating the effects of aging on oocyte maturation and quality. In addition to DNA methylation and histone modifications, the other epigenetic mechanisms including chromatin remodeling, RNA modifications, and non-coding RNA regulation can be intervened to attenuate aging effects. Non-coding RNAs have been demonstrated to play a pivotal role in oocyte maturation by modulating epigenetic, transcriptional, and post-transcriptional events [68]. This regulatory function of non-coding RNAs, which includes endogenous small-interfering RNAs, small nuclear RNAs, and microRNAs, underscores their critical involvement in these critical processes. These non-coding RNAs have been observed to undergo changes in expression in aged mouse oocytes [69]. Consequently, maintaining balanced levels of non-coding RNAs by using suitable interventions during oocyte aging is imperative for preserving oocyte quality at later reproductive ages.

As the genome of an aging oocyte typically exhibits a hypomethylated state [70], manipulation of DNA methylation to bring it into normal levels may be a reasonable approach. Conversely, hypermethylation of specific genes at CpG islands was also observed during aging [71, 72], which should be restored to normal levels. While DNMT1 levels decreased, the levels of DNMT3A and DNMT3B proteins increased in aging oocytes [73]. As it is known, these enzymes are involved in maintenance and de novo methylation processes at CpG islands. It may be reasonable to suggest that restoring the activity of these DNMTs and demethylases in aging oocytes may contribute to reestablishing normal DNA methylation patterns.

### Sirtuin activators

**Fig. 3:**
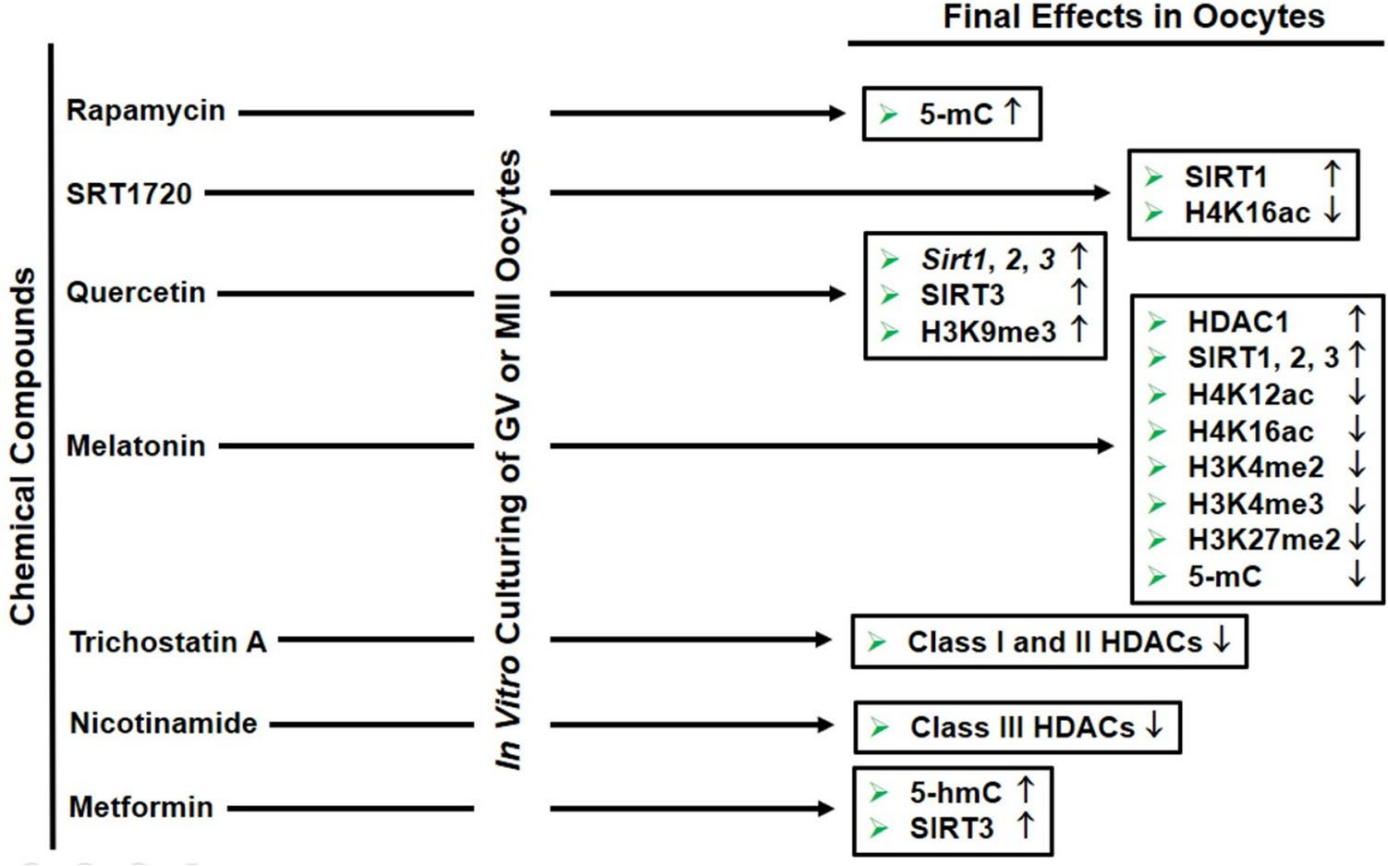
Epigenetic intervention by chemical compound in aging oocytes to regulate DNA methylation, histone methylation, and acetylation as well as related enzymes. Thus, aging-related phenotypes, including meiotic defects, mitochondrial dysfunction, and increased oxidative stress, are mitigated. The icons represent either a decrease in expression (↓) or an increase in expression (↑). 5-mC, 5-methylcytosine; 5-hmC, 5*-*hydroxymethylcytosine; SIRT, sirtuin; HDACs, histone deacetylases; ac, acetylation

The potential of using chemical compounds to regulate epigenetic mechanisms is currently under investigation (Fig. 3). Sirtuin-activating compounds (STACs) are classified as epigenetic drugs due to their potential role as a geroprotector [74]. STACs can induce the sirtuin family of HDACs to regulate histone acetylation accumulation, which may contribute to the prevention of age-related defects due to altered expression of acetylation-related genes. Consistently, SIRT1 activator SRTS has been demonstrated to reduce the incidence of age-related metabolic disorders and enhance the quality of the ovarian reserve, thereby preserving fertility at later reproductive years [75]. As a result, the main purpose of treating these compounds is to balance the spatial and temporal distribution of histone acetylation marks in aging oocytes.

The loss of heterochromatin that occurs during the aging of mouse GV oocytes can be attenuated by activating the heterochromatin machinery via treatment with the SIRT1-activating molecule SRT1720, inhibiting retrotransposon reverse-transcriptase through azidothymidine (AZT) treatment, and overexpressing *Sirt1* or *Ezh2* gene expression [76]. Thus, constitutive heterochromatin is increased, retrotransposon is decreased, and oocyte maturation rates are increased. The SRT1720 also led to decreased H4K16ac level in aged mouse oocytes so that chromosomal segregation errors can be attenuated by the way of increasing the low SIRT1 activity [77]. In addition to the former chemical, the antioxidant N-acetyl-l-cysteine (NAC) has a prominent effect on regulating acetylation accumulation so that it contributes to mitigating adverse impacts of oocyte aging in mice by elevating expression of the sirtuins, especially SIRT1 [78]. NAC treatment during in vitro postovulatory aging of MII oocytes at 18 and 24 h resulted in reduced abnormal distribution of cortical granules and intracellular ROS levels as well as increased intracellular ATP levels and decreased disrupted mitochondrial distribution in mice [79]. Consequently, histone deacetylases such as SIRT1 should be evaluated in aging oocytes. Upon determining its decreased levels, it can be stimulated by treating SIRT1 activating molecules.

The in vivo and in vitro postovulatory aging of mouse oocytes was found to alter the levels of histone acetylation marks such as H3K14, H4K8, and H4K12 [62]. Therefore, it can be posited that HDAC inhibitors have the capacity to reverse the aging-related deacetylation of chromatin [80]. This, in turn, has the potential to result in the activation of stress resistance and longevity proteins.

### HDAC inhibitors

Treatment of aging pig oocytes with trichostatin A (TSA), a HDAC inhibitor, contributed to a reduced percentage of fragmented oocytes and developing to blastocyst stage when compared to the oocytes incubated with TSA-free medium after 24 h aging [81, 82]. As TSA has been identified as an inhibitor for class I and II HDACs, it is essential to evaluate the effects of this inhibition on the accumulation and distribution of histone acetylation marks in aging oocytes.

Administration of the class III HDAC inhibitor nicotinamide (NAM) caused an inhibition of cellular fragmentation, spindle elongation, and formation of astral microtubules in the in vitro aging of mouse oocytes for up to 48 h of culture [83]. Another study by Zhang et al. (2016) found predominantly decreased SIRT1, 2, and 3 levels in mouse oocytes that underwent in vivo or in vitro aging [84]. Reduction of these SIRT expressions was associated with enhanced intracellular ROS levels. Additionally, treating MII oocytes aged in vitro with NAM contributed to increased spindle defects and impaired distribution of mitochondria. These findings showed that reduced SIRT1, 2, and 3 expressions may cause acceleration of postovulatory oocyte aging [84]. It is hypothesized that these effects are the result of altered acetylation of histones and non-histone proteins, including α-tubulin. Taken together, HDAC inhibitors appear to be a potential factor in regulating age-related acetylation accumulation.

### Quercetin treatment

Quercetin, a flavonoid, has been shown to possess antioxidant properties. Quercetin further prevented the decrease in oocyte quality during postovulatory aging (12 and 24 h of additional culturing) in mice [85]. This treatment led to a reduction in aging-related morphological changes and ROS accumulation. Additionally, it ameliorated impairment in spindle organization, mitochondrial localization, and reduced maturation-promoting factor activity and the onset of apoptosis. Additionally, its treatment prevented a reduction in *Sirt1*, *2*, and *3* mRNA levels and H3K9me3 profile [85] (Fig. 3). Quercetin contributed to improving porcine oocyte maturation and reduced in vitro aging effects possibly by increasing expression of BMP1, GDF9, MOS, and CDK2 [86]. Additionally, quercetin decreased intracellular ROS, apoptosis, and autophagy and increased SOD2 and catalase in aged porcine oocytes [86]. In addition to these studies on different mammalian species, further research on human oocytes may elucidate the capacity of quercetin to safeguard the quality at the later reproductive ages.

In a previous study, Cao et al. (2020) reported that treatment of aged mouse oocytes with quercetin resulted in a significant increase in in vitro maturation and blastocyst formation rates [87]. For this purpose, quercetin has been shown to decline ROS through the action of SIRT3, which regulates acetylation at the K68 residue of superoxide dismutase 2 (SOD2) [87]. This flavonoid also reduced in vitro aging effects and improved oocyte maturation following an additional 24 h of culturing [86]. In addition, it decreased apoptosis and autophagy, while concurrently increasing SOD2 and catalase levels in aged porcine oocytes. Indeed, quercetin played a pivotal role in the restoration of defective mitochondrial membrane potential and spindle assembly in aging oocytes [86]. Consequently, quercetin seems to possess the capacity to safeguard oocytes from in vitro aging. This potential protective effect may be attributed, at least in part, to the ability of quercetin to reduce intracellular oxidative stress and enhance mitochondrial function.

### Melatonin treatment

Another epigenetic regulator melatonin is known to function as a scavenger of free radicals, which are produced during the process of oocyte aging. Melatonin treatment to mice at 10 to 43 weeks of age resulted in delaying ovarian aging in mice by rising the actions of antioxidants, telomere maintenance, increasing SIRT1 and 3 expressions, inducing ribosome activation, and decreasing autophagy [88]. In the postovulatory aged mouse MII oocytes, melatonin caused an improvement in the oocyte’s fertilization ability by the ways of reducing ROS levels and inhibiting apoptosis and maintaining localization and levels of the fertilization-linked proteins, ovastacin, and JUNO [89]. As is well-known, ovastacin functions as a CG metalloendoprotease, and it showed mislocalization and premature exocytosis in aged oocytes with disrupted ZP2 structure. On the other hand, JUNO protein is a sperm receptor on the oocyte membrane, and its levels also decreased in aged oocytes [89].

Melatonin treatment also contributed to attenuating the meiotic phenotypes observed in aged mouse oocytes, whether in vitro or in vivo [90]. These phenotypes included spindle and chromosome disorganization, as well as aneuploidy generation. SIRT2 plays a role in mediating the positive effects of melatonin on meiotic structures in old oocytes. In other words, SIRT2 is involved in regulating deacetylation of H4K16, which is essential for maintaining the meiotic apparatus in aging oocytes [90]. Melatonin further regulated HDAC1 expression and the levels of H4K12ac and H3K4me3 in maternal or postovulatory aged oocytes, thereby restoring them into normal levels [51]. As a depalmitoylation enzyme, palmitoyl-protein thioesterase 1 (PPT1) was found to increase in aged mouse oocytes for the purpose of regulating tubulin palmitoylation [91]. Melatonin has been demonstrated to reduce PPT1 levels, thereby contributing to a reduction in PPT1-derived damages. This effect is achieved through the modulation of oxidative stress and lysosomal degradation, which are both induced by PPT1 [91]. All these findings on mice indicated that melatonin plays a role in mitigating the effects of aging on maturing oocytes by modulating various cellular processes, from regulation of oxidative stress to meiotic control.

In the postovulatory aged pig oocytes (for 24 h in vitro), melatonin contributed to mitigating oxidative stress, reducing apoptosis levels, retarding the decrease of mitochondrial membrane potential, and stimulating early embryonic development [92]. Melatonin treatment to prolonged culturing porcine MII oocytes (24 h) was observed to reverse the altered levels of H3K4me2, H3K27me2, and 5-mC, returning them to normal levels [52]. As SIRT1, 2, and 3 are known to decrease in aged oocytes, in vitro melatonin supplementation provided to increase expression of SIRT1 (not for SIRT2 and 3) as well as the antioxidant enzyme MnSOD [93]. Melatonin supplementation to aged bovine MII oocytes further contributed to decreased frequency of aberrant spindle organization and cortical granule release as well as increased mitochondrial membrane potential and ATP production [94]. Additionally, there was an enhanced speed of development of bovine oocytes to the blastocysts following IVF and declined apoptotic index in the blastocyst stage embryos [94] as observed for mouse oocytes [95, 96].

In consideration of the aforementioned findings, melatonin can fulfill a regulatory function in regard to histone deacetylates and the acetylation and methylation marks on histones as well as DNA methylation. This regulatory function serves to mitigate the meiotic phenotypes that manifest in oocytes undergoing the process of aging. The potential effects of melatonin on human aging oocytes should be evaluated in future studies before its routine use is initiated in ART centers. The present results indicate that melatonin delays oocyte and ovarian aging by multiple mechanisms including antioxidant action, mitochondrial function, maintaining telomeres, control of SIRT1 and HDAC1 expressions, ribosome function, and by reducing autophagy.

### Metformin treatment

Metformin is another geroprotective compound that plays a role in extensive epigenetic regulation. It is known to retard the aging process and mitigate oxidative stress. This compound attenuated aging-deriving phenotypes possibly by changing histone methylation levels through altering the ratio of SAM to SAH [97]. Furthermore, metformin has been demonstrated to elevate the levels of 5-hmC modification (showing DNA demethylation process) [98] and microRNA profiles [99]. Similarly, an immunosuppressant rapamycin, a mTOR inhibitor, alleviated the aging-related DNA methylation changes [100]. A recent study reported that the addition of metformin to the culture medium of oocytes from aged mice promoted oocyte maturation and early embryo development [101]. In more detail, metformin decreased meiotic defects and provided normally distributing cortical granules as well as declined apoptosis, promoted mitochondrial function, enhanced the autophagy rate, and reduced mitochondrial ROS levels through SIRT3 action on the acetylation status of SOD2K68 in oocytes from aged mice [101]. Despite the positive effects of metformin on oocyte maturation and early embryo development, its long-term effects on DNA methylation dynamics should be evaluated in future studies, especially on different reproductive and somatic tissues. Subsequently, an evaluation of its potential application in human oocytes is warranted.

### Ectopic expression

The ectopic expression of OCT3/4, SOX2, and KLF4 in aged mice results in alterations to the DNA methylation patterns, which resemble those observed in younger mice [102]. In other words, DNA demethylation, which is stimulated by the expression of OSK, is a prerequisite for the restoration of DNA methylation. Furthermore, OSKM (OSK plus c-MYC) has been observed to remodel the H3K9me3 and H4K20me3 levels, thereby extending the lifespan of progeria mice [103]. The implementation of healthy lifestyle practices, including caloric restriction, regular routines, and moderate exercise, also contributed to the reprogramming of DNA methylation and histone modifications, which ultimately results in the retardation of the aging process [74]. These lifestyle factors may reprogram DNA methylation patterns possibly by altering OSKM levels.

Consequently, manipulation of the epigenetic mechanisms (Fig. 3) may prove an effective means of alleviating the effects of aging, thereby extending reproductive lifespan. It would be beneficial to evaluate cost-effective interventions that regulate DNA methylation and histone methylation/acetylation in mammalian aging oocytes including humans, with the aim of slowing down or reversing these unfavorable alterations.

## Conclusion and future remarks

Aging oocytes at the GV and MII stages exhibit significant alterations in DNA methylation, histone methylation and acetylation status, and the related enzyme levels. These alterations are found to be associated with aging-related phenotypes in oocytes, including reduced quality and quantity, meiotic defects, mitochondrial dysfunction, and increasing oxidative stress. In recent decades, there is a growing interest in exploring exogenous interventions that aim to mitigate the phenotypic effects of these epigenetic changes. While these interventions do not fully reverse the aging process, they have yielded encouraging results, offering potential strategies for safeguarding oocyte quality in the later reproductive ages in certain mammalian species such as mice, bovines, and humans.

The potential reasons of different findings in the studies on different mammalian species including humans may result from distinct aging times and/or evaluating different domains of the same gene. The cumulative effects of epigenetic mechanisms and including variation in microenvironmental factors contribute to the observed divergence among mammalian species. The molecular biological distinctions among mammalian oocytes appear to be associated with distinct reflections of epigenetic changes during their aging and the emerging phenotypes. As expected, interventions to the epigenome of oocytes show different results coming from the aforementioned differences. Thus, a number of detailed studies should be performed on human oocytes to initiate a routine use of these interventions in ART centers for the purpose of increasing reproductive potential at later ages.

Consequently, a considerable body of research in this field has been directed towards the analysis of specific genes and individual epigenetic modifications. However, it is imperative to acknowledge the intricate interplay among epigenetic modifications in the context of regulating transcription and chromatin structure. The interplay between epigenetic modifications and modifiers gives rise to the formation of the epigenome, which exerts regulatory influence over transcription and chromatin structures in a multitude of ways. The future objective is to determine the epigenome of single oocytes with high quality. However, obtaining oocytes from aged mammals poses a substantial challenge, as the use of superovulation can introduce confounding factors that hinder the evaluation of the impact of aging and suboptimal conditions. It would be advantageous to investigate potential epigenetic interventions that could delay or even reverse the impacts of oocyte aging, with the aim of improving fertility in women at later ages and enhancing their reproductive health.

## Data Availability

The datasets generated during the current study are available from the corresponding author on request.
